# The Transition from Childhood to Adolescence: Between Health and Vulnerability

**DOI:** 10.3390/children11080989

**Published:** 2024-08-14

**Authors:** Francesca Mastorci, Maria Francesca Lodovica Lazzeri, Cristina Vassalle, Alessandro Pingitore

**Affiliations:** 1 Clinical Physiology Institute, CNR, 56124 Pisa, Italy; mariafrancescalodovicalazzeri@cnr.it (M.F.L.L.); alessandro.pingitore@cnr.it (A.P.); 2 Fondazione Toscana Gabriele Monasterio, 56124 Pisa, Italy; cricca@ftgm.it

**Keywords:** childhood, adolescence, school, health-related quality of life, health determinants, well-being

## Abstract

Transitioning from childhood into adolescence is an extraordinary time of life, associated with major physical, emotional, cognitive, and social changes and characterized by dynamic development in which interaction with the environment modulates the individual resources responsible for well-being and health. This sensitive period is the time when, in addition to hormonal, metabolic, and neural changes, certain behavioral strategies begin to take shape that will shortly go on to define the emotional, social, and cultural identity of the individual. This narrative review aimed to uncover the crucial processes underlying the transition by identifying processes that are responsible for cognitive, psychosocial, and emotional development, in the absence of disease. For this aim, we highlight (1) the physical, psychological, and social determinants during the transition from childhood to adolescence; (2) the role of health-related variables in resilience or vulnerability mechanisms; and (3) recent school-based strategies to promote health and well-being. Recognizing that health and well-being are the result of the interaction of many biological, psychological, social, cultural, and physical factors will lead to comprehensive health promotion involving all actors joining the growth process, from health professionals and the educational community to parents and community. Furthermore, it is important that psychosocial dimensions are strengthened already during childhood to prevent the onset of frailty and illness in adolescence.

## 1. Introduction

Transitioning from childhood into adolescence is an extraordinary time of life, associated with major physical, emotional, cognitive, and social changes and characterized by a dynamic development in which relation with the environment moderates the individual resources responsible for well-being and health [1]. Although, from a health point of view, adolescence is considered one of the best periods of life, risktendency to underestimate possible risk factors that compromise health status and well-being in the short and long term, predisposing to the onset of disease [2].

This period is the time when, in addition to hormonal, metabolic, and neural changes, certain behavioral strategies begin to take shape that will shortly go on to define the emotional, social, and cultural identity of the individual [3]. It is for this reason that early adolescence has always been regarded as an entirely singular period of life, characterized by the balance between unexpressed potential and fragility [3]. This transition, if not experienced correctly by the child, is the time when vulnerability develops at the expense of adaptive mechanisms that lead to psychological problems, including anxiety, depression, eating disorders, and addictive behavior [4,5]. This interplay between risks and health profoundly affects the resilience and vulnerability mechanism [6]. Although there is a great deal of evidence in the literature on the biological and psychosocial characteristics of childhood and adolescence, to our knowledge there is little that focuses on the delicate transition between one period and the next, especially in the absence of particular diseases; on the contrary, there is a great amount of data with respect to behavioral or other disorders.

To close this gap, this narrative review will allow for a more detailed description of what is known about the transition from childhood to adolescence in healthy children and adolescents, focusing on the physical, psychological, cognitive, and social processes relevant to this complex and multifaceted passage, emphasizing the possible new perspectives to adopt.

## 2. Health Concepts in Children and Adolescents

The concept of health has its roots in ancient Greece and has undergone numerous changes in definition over the centuries. In particular, in the medical–scientific field, there has been a shift from considering health as the ‘*absence of disease’ to a ‘state of complete physical, psychological and social well-being and not simply the absence of disease*’ [7]. While the entire scientific world today agrees on accepting this definition, it is not so simple to assess the true state of health of a population and even less to quantify it, especially in a sensitive period such as the transition from childhood to adolescence. The definition of health for children, and during their transition to adolescence, has been little considered in comparison to that of adults. Although the perspective has evolved from a focus on morbidity and mortality to assess broader aspects of health, such as those of a psychosocial nature, child-/adolescent-specific aspects have been generally excluded. It is important to underline how children’s health, even in the fetal period according to Barker’s hypothesis, has short-, medium-, and long-term influences, with effects both during the transition to adolescence and adulthood [8,9]. Therefore, improving health during childhood, through actions in the different social contexts of reference, school, family, and community, will help to enhance the transition to adolescence, reducing its fragility. According to a new perspective, “*Children’s health is the extent to which individual children or groups of children are able or enabled to (a) develop and realize their potential, (b) satisfy their needs, and (c) develop the capacities that allow them to interact successfully with their biological, physical, and social environments*” [10]. This definition sees health as a positive resource that offers children the ability to interact with the surrounding environment and actively respond to life’s challenges and changes. Furthermore, it considers a fundamental aspect of development: the optimization and maintenance of the state of health itself over time.

Compared to childhood, adolescence is certainly characterized by the greatest criticality, but also by great potential, hence the term “*window of temporal vulnerability*”. During this phase characterized by growth and change, all the body’s systems reset their dynamics and connections, from neuronal to cognitive and emotional functions, motivational systems, and behavior [11]. All these variations, on a biological level, can lead either to a process of resilience, i.e., adaptation to the change that the adolescent experiences, or to processes of instability, in which dysfunctional conduct and risky behavior, such as smoking, alcohol, and substance consumption, take place. This is probably due to a redefinition of neurobiological structures, related to a neurobiological phenomenon, known as ‘pruning’, which determines a neural reset [5]. Precisely due to this new neurodevelopment, mental disorders or, more generally, psychological distress can arise with short- and long-term effects in adulthood [12].

In this complex framework, health promotion and prevention in the transition from childhood to adolescence is a pillar domain, mainly because there is a lack of evidence to consider adolescence as an optimal period of health, but also, according to the Lancet Commission on Adolescent Health, this period is responsible for 35% of the global burden of disease [7,13]. Chronic degenerative diseases, such as cardiovascular diseases, neurodegenerative diseases, and cancer, are some of the most significant public health challenges facing today’s adolescents, aged 10–24 years, who now account for over 25% of the worldwide population. Globally, chronic degenerative diseases are the leading cause of mortality and morbidity in Western countries, with clinical signs usually present during adult age. However, the first changes may occur during the transition, making this period particularly pivotal for implementing preventive interventions. Hence, there is a need to intervene during this sensitive phase, through social and educational contexts, as the benefits take on a connotation of “triple dividend”, referring to children into adolescence, to adolescence when adult, and to future generations [13]. But, when should the preventive educational approach be undertaken? This question is a must considering some experimental evidence according to which approximately half of the pathologies developed in adulthood find their onset during the transition, therefore suggesting that prevention programs in this “time window” of greater susceptibility can significantly reduce the possibility of the onset of chronic–degenerative diseases in future years [2].

## 3. Resilience and Vulnerability in the Transitioning from Childhood into Adolescence

The balance between health and risk factors constantly influences the dynamics of resilience and vulnerability in all phases of life, especially in periods of greater biological criticality such as the transition period between childhood and adolescence.

**Figure 1:**
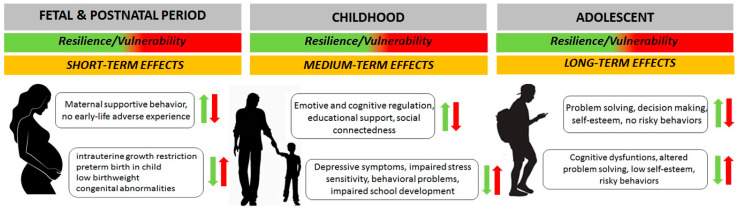
Effects of resilience or vulnerability mechanisms in transition from childhood to adolescence.

Resilience represents the ability of an individual to cope with a traumatic experience or period of allostatic overload. In recent years, the concept of resilience has undergone a change in approach and now is considered a dynamic neural, physiological, behavioral, cognitive, and emotional process that can lead the individual to adapt; on the contrary, vulnerability increases the possibility of developing pathologies as a result of risk exposure. As such, resilience is determined by both extrinsic, primarily environmental, and intrinsic factors such as genes, and by their interactions. A key condition in the resilience process is the simultaneous presence of both risks and promotive factors. Among the risks, recent studies show that it can depend on early experiences during the fetal period [14]. In particular, stressful events in childhood and adolescence such as deficient parenting, poverty, traumatic conditions, natural disasters, and physical illness can predict trajectories of internalizing symptoms in adulthood, hence the importance of learning resilience-building strategies during this period [15,16]. The promotive factors include assets or resources; the first ones refer to coping strategies, learned or innate, competence, and self-efficacy, while the second ones are addressed to the social environment according to the ecological model considering families, services, groups, and community [17]. The association between assets and resources, for example high self-esteem and solid social relations such as emotional support, communication, and modeling, are considered protective against psychological distress, depressive symptoms, and substance abuse because it is related to healthy lifestyle habits [18]. Psychological, biological, environmental, and social factors impact the ability to be resilient or vulnerable during the transition to adolescence. In particular, events during the perinatal, fetal, and postnatal periods impact the child’s ability both during childhood and in the period of fragility at the transition to adolescence to implement adaptation strategies, influencing personality traits, the locus of control, self-efficacy, self-esteem, and cognitive appraisal (Figure 1) [19,20]. Among the various protective factors, maternal care during the perinatal period plays a pivotal role, contributing to a child or adolescent’s ability to cognitively reconsider events and resilience [21].

Within the social context, supportive teacher–student relationships are responsible for good school achievements, better mental health statuses, and low risks of unhealthy behaviors. To this, the use of leisure time such as involvement in extracurricular activities that contribute to positive development, learning social roles, and psychosocial competencies should be added.

Thus, vulnerability appears to play an important role in differentiating multiple modes of adaptation. Vulnerability can also be interpreted as a function that encompasses both personal and social characteristics [22]. This concept is strictly connected with the capabilities to manage negative episodes such as stress or daily life events with a high emotional impact. In particular, young people do not know how to cope with them because they are unable to activate the coping strategies that would allow them to be resilient, while they tendentially activate those maladaptive mechanisms that increase their vulnerability. In other words, adolescents who do not have adequate coping strategies to face the typical challenges of this age might be at risk for long-term pathologies, both physical and mental. On the contrary, when resilient dynamics are available in terms of family life, social involvement, and personal growth, well-being and a healthy transition to adulthood will be potentiated.

## 4. Quality of Life and Well-Being Perception

According to the WHO, health is “*a state of well-being in which the individual realizes his or her own abilities, can cope with the normal stresses of life, can work productively and fruitfully, and is able to make a contribution to his or her community*” [7]. Thus, health is not considered only as the absence of disease, but rather a condition of physical, mental, and social well-being. For this reason, during adolescence, seen as the best period in life, it is correct to not limit it to speaking of health, but rather of a much broader status of perceived well-being, assessed, above all, not so much objectively as subjectively.

Well-being and quality of life have a similar definition, with the first one referred to as “*an individual’s perception of his or her position in life in the context of the culture and value systems in which he or she lives and in relation to his or her goals, expectations, standards and concerns*” [23], and the second one as “*an individual’s perception of their position in life in the context of the culture and value systems in which they live and in relation to their goals, expectations, standards and concerns*” [23]. Thus, they are often used interchangeably.

Actually, these definitions highlight the question of how to quantify well-being and quality of life. Subjective well-being can be divided into three perspectives: evaluative (corresponding to the assessment of life satisfaction), hedonic (corresponding to feelings such as happiness, sadness, grief, and anger), and eudemonic (sense of purpose and meaning in life) [24]. The evaluation of quality of life can thus include objective variables to define the degree of health and subjective variables based on the meaning the individual gives to this. Therefore, there are no real indicators of health and well-being, and the most widely used parameter is health-related quality of life (HRQoL), i.e., the quality of life in the context of one’s health and/or illness. In 2016, the Lancet Commission focused global attention on the health and well-being of adolescents (10–19 years) as key elements in the fields of public health, school-based programs, and preventive medicine [13]. According to this view, HRQoL must include physical, mental/psychological (or emotional), and social health, as well as global perceptions of function and well-being [25]. Therefore, HRQL valuation requires a multidimensional and integrated framework that includes variables belonging to emotional status, cognitive skills, socioeconomic context, and lifestyle habits. However, it is important to emphasize that the concept of HRQL is based mostly on the adult literature and does not allow for developmental changes, language levels, or adolescents’ construction of health and illness.

One tool specifically developed to evaluate children and adolescents’ HRQoL is the KIDSCREEN questionnaire [26,27]. This instrument provides a comprehensive indication of health and well-being perception. The 52 items are grouped into 10 dimensions: Physical and Psychological Well-being, Moods and Emotions, Self-Perception, Autonomy, Parent Relations and Home Life, Social Support and Peers, School Environment, Social Acceptance (Bullying), and Financial Resources [26,27,28].

Another tool to measure HRQoL in children and adolescents is the Personal Well-Being Index School Children (PWI-SC), validated in Portugal and Australia and developed as a cross-cultural instrument to measure the subjective well-being of school children [29]. It is an index of personal well-being that is primarily based on the subjective psychological assessment of mood happiness [29], in accordance with the key point that psychological well-being is a strong indicator of health. To date, in fact, although there are various programs for health promotion and prevention in adolescents, preventive strategies are mainly aimed at adolescents with diseases rather than healthy adolescents. This suggests a fragmentation of services aimed at adolescents.

## 5. Health-Related Dimensions

### 5.1. Lifestyle Habits

Healthy lifestyle habits implemented during the transition from childhood to adolescence are protective against the onset of pathologies later in life [30]. Traditionally, a healthy day should include a high level of physical activity (PA) and thus a low level of sedentary behaviors, a well-balanced diet, and good sleep [30].

Childhood is usually the target for primordial prevention through the promotion of healthy eating habits due to the evidence that the physiological risk of chronic diseases can develop early in life [31]. Diet quality in children could have a lifelong effect on the incidence of several diseases, including obesity, diabetes, heart diseases, and some types of cancer; therefore, enhancing a healthy lifestyle through diet is considered a protective factor [32]. However, if it is easier for children to convey healthy lifestyle habits through the role of family and school, during the transition to adolescence and in adolescence itself, this requires greater attention. In fact, recent evidence shows that adolescents are the group of populations with greater lifestyle habit inadequacy, especially in diet [33].

Given that, it is important to identify what changes in lifestyle habits occur from childhood to adolescence and at what age, to better understand the optimal period to intervene and change the prevalence of unhealthy behaviors. Another important aspect concerns PA, where an association has been demonstrated with health outcomes, well-being, motor skill development, and lower distress [34]. In recent years, a relationship during the transition has also been demonstrated between high PA and lower recreational screen time, supporting, for example, a preventive role of PA in social media addiction [35]. The practice of PA and less sedentary behaviors were associated with higher diet quality and healthy habits during the last year of childhood in cross-sectional studies [36]. On the contrary, sedentary activities in early adolescence are linked to an increase in the consumption of energy-dense foods, soft drinks, and sweets [37]. In this picture, sleep seems to play a central role, as it is considered an important contributor to physical and mental health. In fact, much evidence has shown that better diet quality has been associated with adequate sleep duration, while sleep deficiency during the transition from childhood to adolescence is linked to poor diet [37,38].

Also, sleep quality and duration are associated with emotional regulation, good cognitive functions, and better academic achievement, while poor sleep quality is pivotal to increased food intake and excess body weight [39]. Another important dimension that influences healthy habits is the socioeconomic status condition (SES) [40]. In particular, the perception of living in low SES conditions will increase mental diseases such as general depressive symptoms [41]. In this context, maternal age and educational level are considered predictive of a certain lifestyle; older and more educated mothers are more likely to induce a better lifestyle [42].

Since adolescence is a critical time to define health status, it is pivotal to understand behavioral practice during the transition from early to late adolescence and to adulthood. Studies on adolescents’ healthy habits mainly centered on the relationship between diet and their outcome on health, not considering that sometimes eating problems or diseases originate and correlate with other variables, such as SES [40]. In a previous study conducted in a sample of Italian adolescents, the authors demonstrated that in the female population, physical well-being, mood, and self-perception dimensions were related to weight status, showing that underweight girls exhibited a higher score in these scales as compared to their overweight and obese counterparts. Contrarily, in the male cohorts, BMI categories influenced only physical well-being and self-perception, which were higher in normal-weight boys than obese boys [43,44]. In general, weight-related problems, as consequences of unhealthy behaviors, very often begin during adolescence and continue into adulthood [31]. Intervening in weight alteration directly during this period reduces the possibility that it will also present itself in adulthood as a comorbidity with other pathologies, such as cardiovascular or metabolic diseases [45]. From this point of view, educating young people about a healthy lifestyle is very important, and thus a good diet combined with the practice of PA is needed; in fact, interventions involving both of these aspects for obesity prevention are more effective than single interventions [46]. These results suggest that there is a close relationship between weight status categories and some dimensions of HRQoL, showing how lifestyle habits are affected and influence all other variables, thus suggesting the transversality of preventive strategies to be implemented.

Also, unhealthy behaviors such as smoking, drinking, and substance abuse begin during the transition into adolescence and represent major public health challenges with consequences later in life [47]. However, the long-term consequences of the accumulation of unhealthy early adolescent behaviors on health in later life have been rarely studied. Recent new evidence suggests that dysfunctional behaviors in the transition phase between childhood and adolescence induce epigenetic alterations with subsequent cellular aging, thus suggesting the importance of assessing and modifying unhealthy habits in the period of rapid cell division [48].

### 5.2. Social Context

This period is characterized by a redefinition of the social environment, such as an increase in time spent with peers in addition to family and the structuring of social circles, emphasizing the establishment of a new social and emotional identity [49].

These changes in social effort correspond with a period of marked brain development and refinement in synaptic connectivity and functional integration, the pruning phenomenon, which reflects the development of prosocial behavior, needed for reciprocal relationships [50]. The pruning process leads to a progressive maturation of structures by optimizing space and increasing myelinization, thus making connections more effective. Pruning begins in the dorsal part of the parietal lobe and spreads rostrally towards the frontal cortex. Caudally, it occurs from the parietal lobe to the occipital cortex and ends in the temporal cortex. Some areas, however, of the inferior temporal cortex begin this maturation much earlier, around 4 years of age [51]. This refinement of synaptic connectivity occurs mainly in the areas involved in cognitive modulation, in the strong pleasure-seeking areas, typical of adolescence, and in the processing of social information [52], with influences on well-being perception [53]. In fact, evidence obtained on social networks has shown that the type of adolescent friendship has a significant effect on health, so much so that socially connected adolescents live longer and have greater resistance to various somatic diseases ranging from heart disease to cancer when compared to individuals living in isolation [54,55]. Currently, much evidence reinforces the viewpoint that social determinants are particularly important in the well-being of children becoming adolescents, opening up to the need to assist them in this passage in understanding their health through active involvement in different social contexts.

In particular, growing evidence from education, public health, medicine, psychology, and sociology indicates that well-designed health strategies must fit into the social context in which young people relate. In fact, the perception of having a good social network greatly improves well-being, emotional reactivity, lifestyle habits, and mental health. In particular, support from parents, peers, and school exerted a positive effect [56]. Some previous research studies have shown that the quality of individual social relationships, such as those between parents and peers, is able to go a long way toward modifying the structure, functioning, and development of social regions of the brain in adolescence [56]. Furthermore, it has been seen that among different social settings, family has the greatest involvement, providing the primary structure for a healthy transition. All these findings are in line with the fact that while family social support is protective for healthy behaviors, support from friends is sometimes associated with less engagement in these behaviors [57]. In fact, if in some cases peers can have strong positive influences on adolescents’ health and can be protective against violence and substance abuse, then at the same time, friendship can also increase risk factors such as smoking initiation and persistence, alcohol initiation and use, and sexual risks.

Moreover, in our previous study, we showed how the social context shapes well-being and health status articulated in the different determinants of health, more than any other factor. It is from the social context that the strongest correlations with lifestyle, emotional state, and degree of learning arise [58]. It can be deduced that social context can cover a preventive or predisposing role with respect to chronic degenerative diseases, academic success, professional results, and work performance in the short, medium, and long term [59]. Indeed, a meta-analysis revealed a significant association between social support perception and the development and prognosis of coronary heart disease [60]. Another meta-analysis of 148 studies with more than 300,000 participants showed that people with stronger social support have a 50% greater chance of survival than people with poor or inadequate relationships [55]. On the contrary, social disconnection or poor social relationships, such as isolation and loneliness, enhance vulnerability to health negative outcomes which are added to other adverse events in the course of life [61].

However, evidence from neuroimaging, genetic, and behavioral studies indicates that the social milieu moderates not only psychosocial reactions but also individual differences in brain structure and function. Understanding the mutual interactions between neurobiological and social development may shed light on development pathways linked to risk or resilience for health outcomes; however, the nature of this close and dynamic relationship is still partially unknown.

### 5.3. Emotional Status

The emotional component encompasses all feelings and emotions such as loneliness, sadness, happiness, contentment, resignation, and self-perception. Affective states of both positive and negative nature and also self-esteem are associated with emotional well-being and, consequently, health status [62]. The transition into adolescence is the optimal time to develop positive emotions, needed to build interpersonal relationships, and promote personality, and life skills, reflecting the overall subjective well-being [63,64]. However, the emotions in this period are not stable, probably due to synaptic pruning, mainly localized in the frontal and temporal lobes. Imaging evidence indicates progressive and regressive changes in the volumes of these brain regions, although total brain volume is not significantly altered [65]. The interesting aspect is that the same areas involved in this alteration are also involved in attention, reward evaluation, and goal-directed behavior, suggesting a close connection between emotion and cognition regulation. However, emotional and cognitive development do not correspond to physical maturation. In fact, neuroimaging studies demonstrated that, unlike the adult brain, when faced with an image of fear for instance, the limbic area and the prefrontal cortex, i.e., emotional and judgment centers, are not activated simultaneously, which is how it happens in the adult brain. Such emotional–cognitive asynchrony implies that adolescents have difficulty understanding their own emotions and those of others.

In addition, a study conducted in a sample of adolescents demonstrated that emotions lose stability in this period because they are associated with dynamic and physiological fluctuations [66]. This is an important aspect if we consider that the emotions of adolescents are influenced by genetic, physiological, environmental, and life events and are the results of emotions in childhood [67]. In fact, the capacity for emotional differentiation in the face of ordinary events is lower than in younger children and then in adults. Through the study of emotions, one can gain a lot of information about the person, and this would be very important for educators, teachers, and psychologists in order to adopt preventive strategies best suited to their needs. A recent study by Starr and colleagues in 2019 showed how adolescents who could describe their negative emotions more accurately and precisely than others were less likely to develop depressive symptoms following stressful life events [68]. But one cannot talk about emotions and develop preventive strategies without involving the other determinants of health. In the post-millennial generations, with the spread of devices and social networks, many adolescents have changed their lifestyle habits with a consequent impact on emotional responses. In particular, the abuse of technology has resulted in emotional problems with disrupted social relationships, lower academic achievement, and a lower well-being perception [69,70]. In recent years, much evidence has focused on the role of PA in the emotional modulation of adolescents, demonstrating that PA improves emotion regulation by enhancing brain levels of dopamine, serotonin, and norepinephrine [71]. In addition, a review by Cho and colleagues showed that school physical education program interventions, as compared with other programs in different settings, are important factors in improving positive emotions [72]. Understanding how and why affective responses change with age is not only crucial for characteriszing the emotional development of the child who becomes an adolescent but also for developing appropriate reinforcement strategies and neurobiological development. fMRI studies have shown that the transition from childhood to adolescence is marked by a general decrease in amygdala reactivity suggesting that, unlike children, adolescence is characterized by a shift towards the representation of emotional events in a more cognitive way [73].

### 5.4. Cognitive Functions

Cognitive functions encapsulate activities such as perception, memory, learning, attention, decision-making, language, and thinking. In particular, during the transition from childhood to adolescence, cognitive functions are not yet complete, since the brain areas involved (prefrontal cortex and temporal lobe) have yet to complete their development and are the last to mature; in fact, these are the last areas in which pruning takes place [51]. This is a very important aspect that partly explains the increased transition vulnerability, which is the gap between emotion, cognition, and behavior, with profound consequences on judgment, decision-making, and sensation-seeking [12]. Consistent with evidence on the improvements in reasoning, typical of the passage into adolescence, studies on cognitive functions demonstrate that cognition is linked with the social context in which the subject lives [74]. Social cognitive processes are needed to regulate emotions, manage peer relationships, and develop cognitive abilities. In this field, neuroimaging studies demonstrated that early adolescence appears to be a period of marked neural connectivity between regions involved in sensory and motor functions, while this phenomenon is disruptive in areas implicated in higher-order processes [11]. This suggests that the alteration of connectivity might be functional to the development of more sophisticated neural processes, such as executive functions [75]. An important aspect that correlates with higher functions is sleep and its quality [76]. Sleep is a core behavior during the transition into adolescence, considering that it usually consumes up to a third or more of each day. However, recent studies have highlighted the role of technology, such as devices, smartphones, or tablets, in influencing sleep quality [77]. Already in 2011, the National Sleep Foundation reported that in a survey about sleep habits, more than 95% of the participants used electronic devices in bed or at bedtime, with an impairment of sleep related to cognitive, emotional, and physiological arousal [78]. Associated with this habit, as reported by parents, are behaviors that have been described as bedtime resistance, problems initiating/maintaining sleep, feeling rested upon waking, and an increase in insomnia disorders, although it is poorly featured and under-diagnosed.

Actually, insomnia is considered the main sleep disorder in early adolescence with a strong female prevalence, with profound implications in cognitive and emotional processes [79]. Within cognitive abilities, late-night bedtimes affect academic performances, memory consolidation, knowledge processing, and emotion processing [80]. Scholastic achievement is also compromised by impaired verbal fluency, and reduced problem-solving and creative thought, caused by sleep disruptions [81]. This aspect has consequences in the short and long term, considering that school performance affects health status and well-being. Although it is often thought that education influences health through its effects on future employment and income, without specifying the mechanisms, in reality, the causes of this relationship are to be found in neurobiological and psychosocial dynamics. Educational performance may reflect cognitive abilities and therefore help adolescents to adopt healthier behaviors, but also, on the other hand, students’ health statuses may influence cognitive functions, and thus success in school [82].

Healthy behaviors are strictly related to scholastic success, and this evidence suggests that an unhealthy lifestyle might negatively affect cognitive function. For example, many studies show a link between PA and cognitive function [83]. Recently, a survey involving 2194 early adolescents demonstrated that those who practice moderate PA before mathematics lessons enhanced cognitive engagement [83]. On the contrary, obese students with low levels of PA and healthy habits reported poor academic achievement [84]. Among unhealthy lifestyles, researchers also prove a relationship between cognitive impairments and drug abuse. In more detail, nicotine exposure during neurodevelopment induces long-term deficits in contextual learning, while cannabis use is associated with low academic performance [85,86]. Hence, considering that health status is associated with healthy lifestyles, eating well, getting quality sleep, adapting to stressful events, abstaining from drug abuse, and cultivating good social relations, it is pivotal to implement multi-sectoral, cross-cutting strategies for the various determinants of health, correlating multiple variables in order to decrease the risk of illness and increasing well-being perception.

## 6. School-Based Health Promotion Programs

Providing children in the transition to adolescence with coping skills and protective behaviors can help them to react positively to life’s changes and pitfalls, enabling better mental, social, and academic success. Considering that each dimension of health interacts, the management of one also involves the others, and a beneficial effect on, for example, lifestyle will also have positive consequences on emotional, relational, and cognitive levels [5].

In particular, preventive interventions are possible when actions are directed at individuals, families, and at schools, creating a health-promoting environment.

To our knowledge, many programs are aimed at the management of certain behavioral disorders, while those aimed at healthy children/adolescents are very few [87,88,89,90]. Interventions aimed at the healthy individual are mainly directed at improving or enhancing lifestyle, with programs aimed at controlling nutrition, physical activity, and reducing risk behaviors [91,92,93,94]. In recent years, many school-based programs have addressed the management of bullying, with a reduction in bullying victimization of 20–30% [95]. The AVATAR project is a school-based program based on a multidisciplinary team that includes researchers, teachers, schools, relatives, and institutions, willing to collaborate and cooperate with each other in order to correct unhealthy behaviors, and at the same time, enhance adolescents’ strengths through personalized interventions, not only at an individual level, but also at a school and family level [58,96,97]. In general, all these projects emphasize that schools should be a focal point for prevention, and interventions should involve multiple social components and include the home environment. According to this perspective, WHO suggests the need for an integrated and holistic approach to health and well-being, focused on the possibility of dealing with emotions, social relationships, physical changes, and all different frailties not yet of clinical value, but which could become so.

This holistic approach, which implements health education in the environment and philosophy of the scholastic setting, moves beyond individual behavioral change to improve the social environment with particular attention to its curricula and learning methods.

The strategies promoted by WHO are mainly concerned with mental health and emotional control, to the extent of publishing, in 2022, the guidelines in “Mental Health: Strengthening our response” [98]. Although the proposed activities are heterogeneous, they are all addressed to schools, considering it is the primary setting where such social–emotional learning occurs. School and educational context have a significant role in health and well-being promotion, creating a healthier environment not only for students but for the whole community, considering them additional or alternative to healthcare settings.

Besides the traditional school health guidelines for promoting healthy eating and PA, including the coordination of school policies and practices, inclusive environments, nutrition services, and physical education, in recent times, interventions focusing on stress reduction have become more widespread. According to this perspective, such strategies, e.g., based on mindfulness, which aims to lower both physiological and psychological activation thresholds in order to improve individual skills to deal with stress of different origins, can represent an effective counterbalance capable of reducing behavioral risk factors [99]. Many studies in this field demonstrated that school-based programs targeting anxiety and stress through coping skills, and relaxation techniques, reduce physiological arousal, internalizing problems, and increase self-esteem and cognitive abilities [99]. These outcomes were also shown by applying mindfulness programs and promoting emotional skills, especially in secondary school students [100]. However, although the presence of these encourages promotion and prevention programs, many protocols and much evidence are addressed towards adolescents with disease and not towards the healthy population for prevention purposes. In addition, the policies of different governments are highly fragmented and poorly coordinated. For these reasons, a multifaceted approach is needed that considers socioeconomic, cultural, and environmental factors.

**Figure 2:**
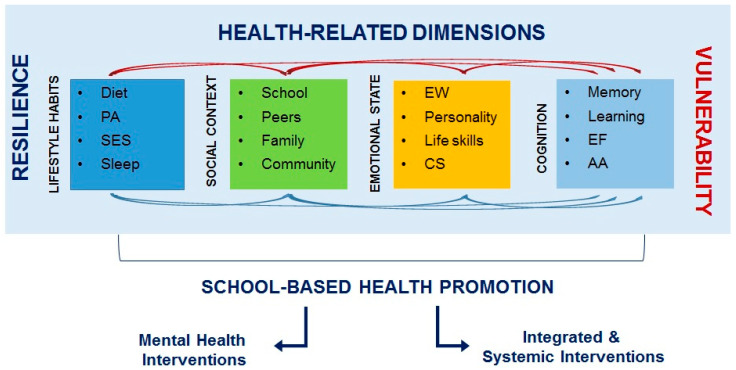
A comprehensive and multilevel approach to health in children and adolescents. PA: physical activity; SES: socioeconomic status; EW: emotional well-being; CS: coping strategies; EF: executive functions; AA: academic achievement.

A comprehensive approach to health at school, with a wide range of activities taking place in schools and in their community, would accompany children during their transition to adolescence by improving their health, developing their potential, and cultivating appropriate relationships (Figure 2).

## 7. Conclusions and Future Directions

This narrative review aimed to uncover the crucial processes underlying the transition from childhood to adolescence by identifying processes that are responsible for cognitive, psychosocial, and emotional development.

This review describes the paradox between health and vulnerability with implications for future strategies to adopt in the school and in other educational contexts [101,102]. This paradox is essentially related to the fact that during early adolescence, typical risk factors begin to emerge, including substance use, different types of addiction, and unstable social relationships, which may contribute to creating fragilities that over time become real disorders. It is no coincidence that approximately 75% of adult mental health disorders first appear before the age of 24 [103]. This dualism is profoundly linked to the resilience or vulnerability phenomenon, considered a multifactorial process including genetic, epigenetics, and coping skill factors. External resources such as the social environment, which plays a key role in modulating development, are also involved in this mechanism. In fact, in order to be able to develop healthy, non-dysfunctional behavior, it is already necessary to develop those behavioral strategies during childhood that enable adaptation, essentially enhancing the social context of reference and prosocial behavior as predictors of resilience.

In line with the pivotal role of social context, the findings reviewed here highlight how during the transition we observe fundamental changes in the social environment, in terms of different educational settings, number of peers, and time spent outside the house with consequences on emotion regulation and cognitive function intended as scholastic achievement. Second, this review describes the different variables and factors that affect the health and well-being of subjects, no longer children or even adolescents already neurobiologically developed, emphasizing how the fragility of one of these influences everything else. Children and adolescent health promotion is a key component of public health, school health programs, and preventive medicine in view of the primordial prevention to reduce chronic degenerative disease later in life. A further challenge will be to involve not only schools, teachers, and students in this process, but also all institutions working in the field of education, wellness, and health promotion [104]. Although this commitment to the health of adolescents has achieved some progress, there remains a need for more innovative action in healthy subjects. In this context, the need to engage adolescents in planning and decision-making processes that will affect their health and well-being perception, now and in the future, must lead to a transition from a clinically oriented system to an education-based model that considers subjects in all aspects of experience and in different social contexts, in order to avoid the consolidation of dysfunctional behavior that will lead, during adolescence, first to fragility and then to pathologies [105,106]. In this context, a personalized approach may facilitate the participation of adolescents and children in educational programs, also making them direct actors in these programs. Recognizing that health and well-being are the result of the interaction of many biological, psychological, social, cultural, and physical factors will enable the adoption of strategies aimed at comprehensive health promotion. Therefore, future challenges should be aimed at defining programs that take into account the different environmental and social settings in which individuals live, also taking into account economical, geographical, and political differences, but targeting healthy individuals to enhance those biological and behavioral traits that are mainly involved in the transition from childhood to adolescence.

## Data Availability

No data were used for the research described in the article.
